# Valuing a Reduction in the Risk of Chronic Kidney Disease: A Large-Scale Multi-Country Stated Preference Approach

**DOI:** 10.1017/bca.2024.16

**Published:** 2024-09-11

**Authors:** Chris Dockins, Damien Dussaux, Charles Griffiths, Sandra Hoffmann, Nathalie Simon

**Affiliations:** 1 U.S. Environmental Protection Agency, Washington, DC, USA; 2 OECD Environment Directorate, Paris, France; 3 USDA Economic Research Service, Washington, DC, USA

**Keywords:** kidney disease, health risk, health valuation, stated preference, willingness-to-pay, D61, I18, J17, K32, Q51, Q53, Q58

## Abstract

Compromised kidney function is associated with an array of environmental contaminants and pathogens that may be considered for regulation. However, there are few valuation estimates for kidney effects for use in benefit–cost analyses, particularly willingness-to-pay estimates. This paper is one of several surveys valuing morbidity developed by the OECD Surveys to elicit Willingness-to-pay to Avoid Chemicals-related negative Health Effects project, which aims to improve the basis for benefit–cost analyses. We report the results of a stated preference survey valuing reduced the risk of symptomatic chronic kidney disease, filling an important gap in the valuation literature and addressing a need for applied benefits analysis of chemical regulation. The survey was administered to representative samples in each of 10 countries: Canada, Chile, China, Denmark, Germany, Italy, Norway, Türkiye, the United Kingdom, and the United States. The mean (median) WTP for an average reduction of 3.5 in 1,000 of the risk of serious kidney disease over 5 years is $2,609 ($764), corresponding to a mean (median) value per statistical case (VSC) of chronic kidney disease of $805,000 ($224,000). The mean VSC varies between $700,000 for Canada and $1,200,000 for Türkiye.

## 1. Introduction

Governments regularly conduct benefit–cost analyses to help policymakers understand the merits of major policy proposals that affect mortality and morbidity risk. While there is a rich literature on willingness to pay (WTP) to reduce mortality risk, it is widely recognized that there are relatively few studies on willingness to pay to reduce the risk of non-fatal outcomes (Cameron, 2014). To address this gap, the OECD has been implementing a coordinated set of stated preference surveys, the Surveys on Willingness-to-pay to Avoid negative Chemicals-related Health Effects (SWACHE) project.^1^

We present the results of a stated preference study, implemented in 10 countries, estimating WTP for reduced risk of serious kidney impairment, defined as stage 3 and 4 chronic kidney disease, the serious chronic phase of kidney disease that is followed by permanent kidney failure (end-stage renal disease (ESRD)) in about 35 % of cases. Serious kidney impairment has been linked to exposure to several chemicals, including heavy metals, certain organic solvents, polycyclic aromatic hydrocarbons (PAHs), and biotoxins (Kataria et al., 2015). Kidney effects have also been associated with exposures to “GenX” chemicals (a trade name for a technology used to make high-performance fluoropolymers) and perfluorobutane sulfonic acid (PFBS) (U.S. EPA, 2018). In addition, some foodborne or waterborne biological organisms, e.g. Shiga toxin-producing *E. coli*, can produce biotoxins that can cause serious kidney damage (Obrig, 2010).

Few studies have estimated WTP to avoid or reduce the risk of chronic kidney disease. Herold (2010) conducted a stated preference survey of U.S. patients with permanent kidney failure (or ESRD) to explore factors influencing their WTP to secure a kidney for transplant, reporting non-zero WTP ranging from less than $2,000 to over $50,000. Kjaer et al. (2013) used a discrete choice experiment of 206 respondents to estimate WTP to establish nephrology treatment facilities in Greenland for patients with permanent kidney failure.

The European Chemicals Agency (ECHA) (2014) estimated WTP to avoid acute kidney injury and WTP to reduce the risk of permanent kidney failure (ESRD) in the Czech Republic, the United Kingdom, the Netherlands, and Italy. Using a chaining approach (Carthy et al., 1998), WTP to avoid ESRD was estimated at less than $5,000. The authors suggest estimates be “treated with caution” in part because responses do not seem to be responsive to illness length or severity.

Rigby et al. (2017) estimated WTP to avoid permanent kidney failure in adults and children and found WTP per statistical case of about $80,000 and nearly $250,000, respectively. As with the ECHA study, the survey does not consider the longer and more common phase of chronic kidney disease that usually precedes permanent kidney failure.

These studies estimate WTP for an aspect of kidney disease (obtain a transplant or avoid kidney failure), but there appear to be no studies that provide WTP estimates for serious chronic kidney disease generally and that produce results applicable to valuing the reduction in risk of serious chronic kidney disease and can be used for benefit–cost analysis. The results here are the first WTP values for kidney disease that are broadly applicable for benefit–cost analysis, available for several countries and internationally comparable.

## 2. Survey design

We estimate values for symptomatic kidney disease, including specific impacts from disease progression, a more broadly applicable endpoint for valuing the impacts of chemical and biotoxin exposure that can cause kidney disease. Specifically, we value the risk of stages 3 and 4 chronic kidney disease, labeled as “serious kidney disease,” and the probability that it progresses to kidney failure (stage 5).

The survey presents an accurate but succinct description of chronic kidney disease, its impacts, and its causes. The survey explains the symptoms associated with serious kidney disease (e.g. lower back pain, nausea, difficulty concentrating) and how kidney disease can lead to other major health complications (e.g. high blood pressure, cardiovascular disease, fluid in lungs, weakened ability to fight infections). The survey description also includes the 35 % likelihood that serious kidney disease can lead to permanent kidney failure and provides a simplified, but detailed, description of what is involved in being on dialysis, the chances of finding a donor kidney, and lifestyle changes and medical care needed following a kidney transplant. The description of kidney disease concludes with information on the impact of serious kidney disease on life expectancy.

Early pretesting made it clear that a scenario based on treatment was not viable because it raised questions about why the treatment was not covered by existing public health programs in many countries, and how the elicited payments related to those programs. Consequently, the survey relies on a scenario where the mechanism for reducing risk is not specified, but the costs are clearly out-of-pocket expenses. This approach has been used successfully in prior stated preference surveys (Krupnick et al., 2002; Hoffmann et al., 2017). Respondents were informed that only the risk of kidney disease would be affected and that no other effects would occur.

We designed the survey instrument through a multi-step process followed by all surveys in the SWACHE project. This started with a description of the health endpoint and an evaluation of how to characterize the endpoint in the survey, followed by the development of the risk reduction mechanism, the payment vehicle, and the elicitation method. A steering group of experts, including academics, BCA practitioners, regulators, and health professionals, provided regular feedback throughout the process.

The draft survey was initially tested in 51 one-on-one interviews across Chile, Denmark, Italy, and the United States. The near-final version of the survey instrument was translated into the necessary languages for all countries, with the translations verified by native speakers, and subjected to 50-person pilot tests in each country prior to it being fielded.

The survey uses a double-bounded dichotomous choice (DBDC) question with an open-ended follow-up question. Risks are characterized as chances per thousand over 5 years, displayed numerically and graphically, together with a short tutorial on risk, risk changes, and how these are presented in the survey. Aggregating risk over 5 years was necessary for risk changes to be plausible and effectively communicated to respondents given baseline risk levels for kidney diseases, particularly for the youngest age group.

Respondents for each country were divided into three age groups (18–40, 40–60, and over 60) to establish the baseline risk of serious kidney disease (15, 25, and 60 in 1,000).^2^ Members of each age group were randomly assigned into one of two hypothetical risk reductions (2 in 1,000 and 5 in 1,000) and one of four possible starting bids. The follow-up bid for the second dichotomous choice question was the first bid multiplied by either two or one-half, following conventional approaches as described in Carson and Hanneman (2006).^3^

The bid structure is shown in Table 1.

**Figure 1.**
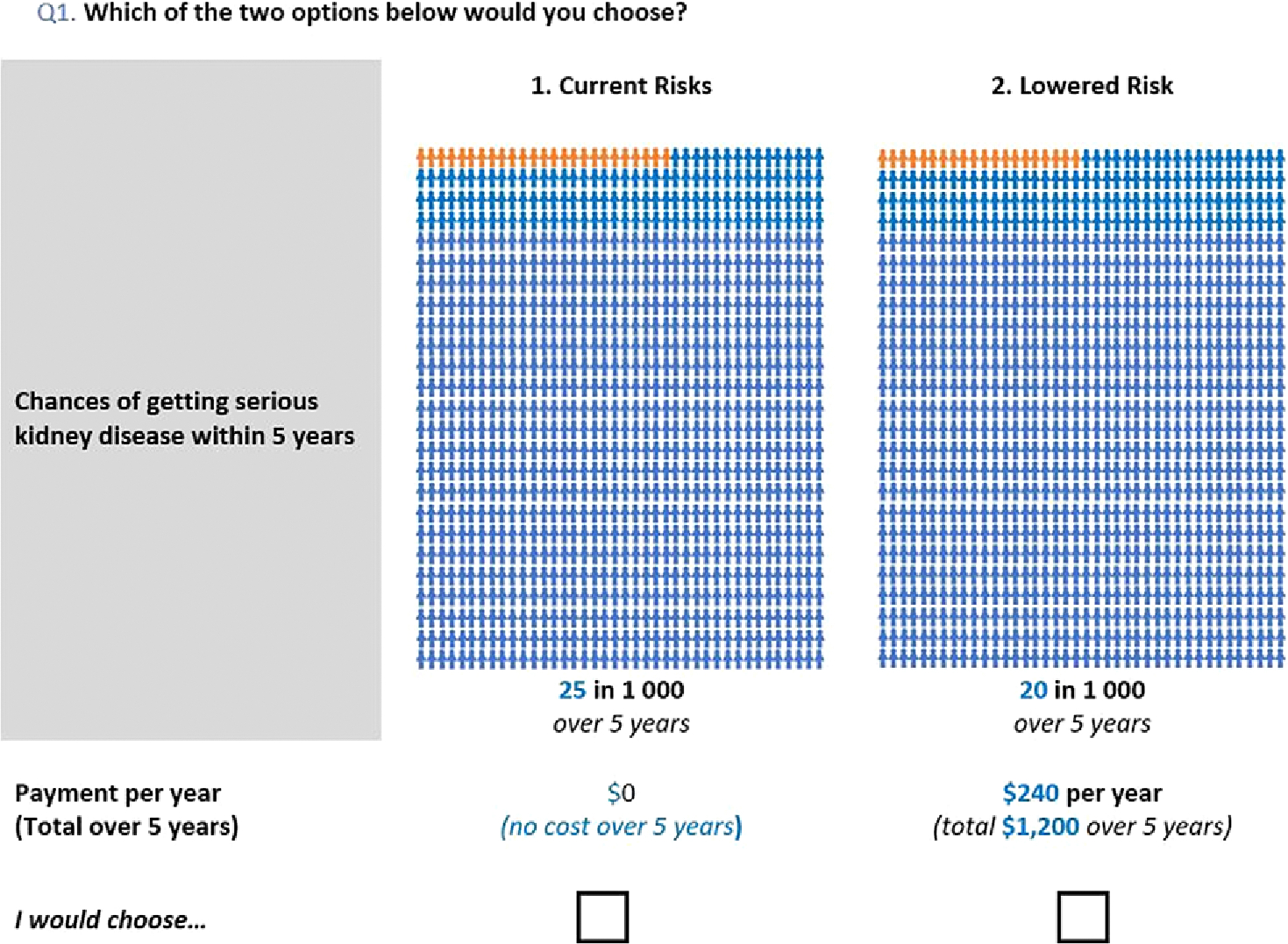
Sample WTP question.

The payment is stated as both an annual figure and as a total over the 5 years. An example of the valuation question is shown in Figure 1.

After the risk tutorial, 91.5 % of respondents correctly answered a simple test employing the risk communication device in which they were asked to identify the scenario with the highest probability of occurring.

All surveys in the SWACHE project share a common set of debriefing questions to identify common issues of concern. Key questions include whether respondents understood that the risk reductions were to occur only over the 5 years described in the survey and were not permanent, and whether they considered benefits beyond those described in the scenario.

## 3. Data

The survey was administered between June 2021 and June 2022 to a sample drawn from a large panel of individuals maintained by Ipsos, a global marketing and survey research firm. Representative samples were drawn for each country based on quotas matching key country-specific demographic characteristics: gender, age group, level of education, and geographic region. Respondents were screened to exclude those with chronic kidney disease. A total of 14,641 individuals started the survey after passing the screening questions, and 12,614 finished the survey. Ipsos removed 614 respondents due to a low-quality response score based on survey speeding (completing the survey in less than 1/3rd of the median survey time), straight-lining, and the proportion of “don’t know” answers, leaving a sample of 12,000.

The data were then screened based on core principles for empirical analysis agreed upon by the SWACHE researchers.^4^ Screening included removing individuals who failed the survey’s probability test (8.47 %) and “speeders” who completed the survey or valuation questions in less than 48 % of the country-specific median time of the sample (12.4 %).^5^ This screening is in addition to the screening done by Ipsos, and is based on the recommendations of Survey Sampling International (2013) and Mitchell (2014), and reduced the sample to 9,709 observations. Additional factors that might affect WTP were also identified, including gender, monthly household income, level of education, health expenditure paid for out of pocket, higher or lower average perceived health, having a friend or relative who experienced kidney failure, being diagnosed with some other chronic disease, and being diagnosed with or having a friend or relative diagnosed with COVID-19. These additional factors were used as explanatory variables in the econometric analysis.

Respondents were asked to indicate their household’s monthly income after income taxes had been paid and were presented with 10 income ranges corresponding to income deciles in their respective countries. Income deciles correspond to equivalized income. Unequivalized income deciles are derived by multiplying equivalized income deciles in 2019 from OECD Income (IDD) database by the number of “equivalent adults” using data on family composition from OECD Family database.^6^ Respondents who did not indicate their range for the income deciles were presented with bigger ranges corresponding to income quintiles in their respective countries. Most respondents (91 %) provided information about the total income of their household. Income ranges were then converted into a single amount to facilitate the use of income data in the empirical analysis. For the smallest income range between 0 and the first decile, the income is set equal to 0.5 times the first decile. For the largest income range above the last decile, the income is computed as equal to 1.5 times the top decile. For all the other income ranges, the computed income is the simple average between the two deciles. All income values were then converted into purchasing power parity adjusted U.S. dollars (USD PPP) using PPP for actual individual consumption data for 2019 from the PPPs and exchange rates OECD database.^7^

We imputed missing income responses by performing country-level Ordinary Least Square (OLS) regression analyses of logged income as a function of indicators for age, couples, females, high education, number of people in the household, employment, parttime work, and whether the respondent was retired.

Table 2 presents the sampling target and the achieved sample for target characteristics of the screened sample. The table shows an over-representation of females in all countries except Denmark, Norway, and Türkiye. This over-representation was present in the full sample received by Ipsos but was larger after our screening. The achieved sample is close to the sample target for age groups. China and Türkiye show an under-representation and Demark an over-representation of individuals over 60. Low and medium levels of education were collapsed into a single group because of an under-representation of lower-educated respondents in all countries. Higher educated respondents are close to the target except for Norway and Türkiye.

A respondent’s monthly income is the average of the income range selected, converted to USD PPP. The median monthly income for Canada, Denmark, Italy, Norway, and the United States closely matches the population median. The achieved sample for Chile, Germany, and the United Kingdom show a slightly higher deviation, with an under-representation of low-income individuals. The two outliers are Türkiye and China, which show much higher incomes in the achieved samples than the population medians. The sample median income is 162 % higher than the target for Türkiye and 227 % higher for China.

Given that the Chinese sample has a median income of more than 225 % of the Chinese population median and that 28 % of the Chinese respondents completed the survey and valuation question exceptionally quickly, China was removed from the baseline analysis, but is included as a sensitivity analysis in the main results table and in the country-level parametric estimations of WTP.

To test for sensitivity to scope and scale, Tables 3 shows the response to the first dichotomous choice question is broken down by the risk reduction offered and the starting bid. For presentation purposes, the starting bid is presented as the 5-year cost for U.S. respondents, as described in Table 1. In practice, the starting bid is a continuous variable because the U.S. dollar values were rounded after being converted to the local currency. For both risk reduction values, the per cent of respondents who answered “Yes” to the first question declines with the starting bid, indicating that respondents are less likely to be willing to pay for a risk reduction as the bid increases. This is the expected sensitivity to scale. Additionally, for the three starting bids that are common to both risk reduction values, the per cent of respondents who answered “Yes” is higher for the larger risk reduction. This suggests that respondents are willing to pay more for a larger risk reduction, which is the expected sensitivity to scope.

Tables 4 and 5 display the summary statistics for the baseline sample of 8,905 respondents who remained after screening those who failed the probability test or were identified as speeders and after removing China. Table 4 provides the summary statistics for the important continuous variables used in the analysis. The first block of data (5 rows) shows the time to complete the survey and the time respondents spent on the two dichotomous choice questions. Respondents answered the second risk question twice as fast as the first one, probably due to increased understanding after answering the first question. The second block of data shows the bid values for the first and second dichotomous choice questions, with the second bid being either one-half or twice the first bid value. Twenty-five per cent of respondents chose the lower risk (responded “yes” to pay the cost) for both risk questions, and 40 % of respondents chose the current risk (responded “no” to pay the bid price) for both questions.

The last rows in Table 4 provide statistics for baseline risk and income, continuous variables considered to be important factors affecting willingness to pay. The baseline risk is 15, 25, or 60, depending on the age group. The PPP-adjusted reported median income ranges from $263 (in Chile) to $17,552 (in the U.S.). Almost 10 % of the baseline sample declined to report their income, but the income with the imputed values used for the missing responses produces a very similar income distribution and provides 8,905 observations for the estimation.

Table 5 provides the summary statistics for the indicator (0/1) variables used in the analysis. The table reports the number of observations for which the variable is equal to one, and the per cent of the total 8,905 observations. The first block on the left-hand side reports the observations for each country after screening for speeders and those who failed the probability test. China is not included in this list because it was removed from the baseline sample. Denmark, Türkiye, and the U.S. have a slightly lower per cent of the observations because of a larger number of speeders and respondents who failed the probability test. The second two blocks on the left-hand side of Table 5 show the age–gender distribution of the baseline sample. As noted previously, there is an over-representation of females in this sample.

The first block on the right-hand side of Table 5 shows the distribution of responses to both risk questions. Twenty-five per cent of respondents chose the lower risk (responded “yes” to pay the cost) for both risk questions, and 40 % of respondents chose the current risk (responded “no” to pay the bid price) for both questions. Approximately 15 % and 20 % of the respondents answered “yes-no” and “no-yes” to the first and second dichotomous choice questions, respectively.

The second block on the right-hand side provides statistics for the indicator variables considered important factors affecting willingness to pay. A large per cent of respondents (38.7 %) believe that their health is better than average, while 15.9 % self-report lower than average health. The majority of respondents (65.8 %) have never been diagnosed with a chronic disease, but over a third (34.8 %) had a relative or friend with kidney disease. Only 8.5 % of respondents report having been diagnosed with COVID-19, but almost 40 % have a close friend or relative who had been diagnosed with COVID-19.

The final block on the right-hand side of Table 5 contains the statistics for controls used to test the robustness of the screening analysis. 5.8 % of the respondents reported that they thought the risk reduction was permanent. 9.2 % considered changes in other health issues not described in the survey (co-benefits) when they made their choices. Eleven per cent of the respondents strongly agreed (on a 5-point Likert scale) with the statement that they would pay almost any amount to reduce risks to their health. Combining those who strongly agreed to pay almost anything with the 25 % of respondents who answered Yes-Yes to the two dichotomous choice questions produces 6.2 % of respondents classified as yea-sayers. 3.4 % of respondents reported that they did not answer as they would have in real life or said that the survey did not provide them enough information to make informed choices. These respondents were classified as protesters.

## 4. Estimation model

To derive mean and median WTP estimates for a reduction in the risk of serious kidney disease, we employ a maximum likelihood estimator using the binary outcomes of the double-bounded dichotomous choice.

We assume a Weibull distribution for the utility error as our baseline because it generally has a shorter right tail than the log-normal and, in its “spike” configuration, usually performs well (Kriström, 1997; Carson & Hanneman, 2006).

We employ a spike model to account for the possibility that a small share of the population might still choose the status quo even if it costs them nothing. This spike could be significant in the case of serious kidney disease because the baseline risk is relatively small for younger respondents. Carson and Hanemann argue that failing to include a spike parameter can, in some cases, lead to overestimating WTP.

We measure the spike by using responses to the dichotomous question and responses to the open-ended question that followed the double-bounded dichotomous choice: “What would be the most you would be willing to pay, if anything, to reduce your chance of getting serious kidney disease within 5 years?”. The spike dummy variable equals one when respondent i chooses no to both valuation questions and responds with a value of zero to the open-ended questions.

A Weibull distribution θ={k,λ} is characterized by a shape parameter k and a scale parameter λ. All estimations assume a shape parameter equal to 1. The baseline specification of the scale parameter when b>0 is

(1) $$\lambda_{ic}(b)=\alpha_{0}+\alpha_{1}\Delta R_{i}+\alpha_{2}lnb_{i}+\sum_{c}\delta_{c}\left(d_{ic}\times \omega_{i}\right)$$

where $\Delta R_{i}$ is the risk reduction proposed to respondent $i,\;lnb_{i}$ is the logged cost or bid proposed to respondent $i,\;d_{ic}$ is a country dummy equal to 1 when respondent i lives in country c, and $\omega_{i}$ is the post-stratification weight of respondent i. Including post-stratification weights, $\omega_{i}$, as a control allows us to capture the fact that some categories of people were slightly under- or over-represented in the sample compared to the actual population.^8^ The more respondent i is underrepresented in the sample, the higher his weight $\omega_{i}$. It is necessary to interact country dummies with the weights because the weights are defined at the country level.

The spike parameter when b=0 is

(2) $$\eta_{ic}=\alpha_{0}+\alpha_{1}\Delta R_{i}+\sum_{c}\delta_{c}\left(d_{ic}\times \omega_{i}\right).$$

The model is also estimated when the scale parameter includes additional explanatory variables as follows:

(3) $$\lambda_{ic}(b)=\alpha_{0}+\alpha_{1}\Delta R_{i}+\alpha_{2}\;lnb_{i}+\sum_{c}\delta_{c}\left(d_{ic}\times \omega_{i}\right)+\alpha_{3}{\text{Female}}_{i}+\alpha_{4}\;lny_{i}\;\;+\alpha_{5}{\text{HighEduc}}_{i}+\alpha_{6}{\text{Baseline}}_{i}$$

${\text{Female}}_{i}$ is a dummy variable equal to 1 when respondent i identifies as a female, $lny_{i}$ is the logged monthly income for the household of respondent $i,\;{\text{HighEduc}}_{i}$ is a dummy variable equal to 1 when respondent i achieved high education outcome, and ${\text{Baseline}}_{i}$ is the baseline risk presented to respondent i.

The model is also estimated when the scale parameter includes information on other important factors. These factors include whether respondents must pay for health expenditures out-of-pocket; whether they perceive their health as below or above the average of people of their gender and age; whether they know a relative who had kidney failure; whether they are diagnosed with any other chronic disease; and whether they or a relative was ever diagnosed with COVID-19.

The mean WTP for an average 3.5 in 1,000 risk reduction in serious kidney disease is computed as a simple average of the individual mean WTP follows:

(4) $$\hat{WTP}=\frac{1}{n}\sum_{i=1}^{n}{\hat{WTP}}_{i}$$

The individual mean WTP is computed by integrating the probability of responding yes to the valuation question over the interval from 0 to maximum bid with adjustment:

(5) $${\hat{WTP}}_{i}=\int_{0}^{b_{max}}\frac{f\left(\lambda_{ic}(b),k\right)}{1-f\left(\lambda_{ic}\left(b_{max}\right),k\right)}db$$

where f is the density function of the Weibull distribution and k denotes the shape parameter. Truncation at maximum bid level $b_{max}$ is necessary since the right tail is not null when the cost goes to infinity. The adjustment of the denominator compensates for the fact that the support of $f\left(\lambda_{ic}(b),k\right)$ does not stop at $b_{max}$. The median WTP is computed as a simple average of individual median WTP as follows:

(6) $${\tilde{WTP}}_{i}=\frac{ln2}{\left|\alpha_{2}\right|}e^{\eta_{ic}\left(\frac{1}{\left|\alpha_{2}\right|}\right)}$$

$\alpha_{2}$ is the parameter for the logged bid value as indicated in equation (1).

Mean and median WTP are also presented as the value of a statistical case of kidney disease avoided. VSC is calculated using the standard method of dividing annual WTP by the average annual risk reduction. Because this survey asks about risk changes over a 5-year period and payments over this same period, VSC can equivalently be calculated as 5 *x* annual WTP divided by the average 5-year risk reduction of 3.5 per 1,000.

## 5. Results

The parametric estimation results of the dichotomous choice model are presented in Table 6. Column 1 shows the baseline estimation results. The size of the risk reduction has a positive and statistically significant effect on the joint probabilities of choosing the reduced risk options, indicating scope sensitivity. Consistent with expectations, the additional cost of choosing the reduced-risk option has a negative and statistically significant effect on the likelihood that it is chosen. The spike variable equals 0.035 and is statistically different from zero. In other words, the average probability that people are indifferent to the valued item is 3.5 % of the estimation sample. This spike at zero is small but high enough to justify using a spike model. For an average reduction of 3.5 in 1,000 in the risk of serious kidney disease over 5 years, mean WTP is $2,609 and median WTP is $764. The mean value of a statistical case (VSC) of serious kidney disease is $805,000 and the median VSC is $224,000.

These results are robust to alternative methodological choices. Column 2 shows the estimation results when the post-stratification weights and its interactions with country dummies are not included as regressors. Column 3 excludes the possibility of a spike at zero. Column 4 assumes a log-logistic distribution rather than a Weibull distribution, while column 5 assumes a log-normal distribution. Column 6 includes survey responses from China, and column 7 excludes survey responses from Türkiye. China was excluded from the baseline model for the reasons described above. Türkiye was excluded in column 7 because the median income of respondents from Türkiye is twice as high as the median income of the population of Türkiye. All columns show statistically significant scope sensitivity and a negative impact of cost on the joint probabilities to choose the reduced risk option that is statistically different from zero. The mean VSC varies from $698 (without a spike) to $1,093 (using a log-normal distribution), and the median VSC varies from $176 (from the log-normal model) to $373 (when including China). The largest deviation of the mean WTP from the baseline estimate is when a log-logistic and log-normal distribution is used, and when survey responses from China are included in the estimation sample. The Weibull distribution was chosen because of a lower AIC score, and as explained previously, the Chinese respondents in this sample are richer than the actual Chinese population.

Table 7 shows the baseline model with likely determinants of WTP added as additional cofactors. The statistically significant determinants are the size of the risk reduction, income, and not having another serious disease. Below average health is marginally significant at the 5 % level. The cost has a negative effect on the probability of choosing the reduced risk option, and income and the size of the risk reduction have positive impacts. Not being diagnosed with another serious disease has a negative effect on the probability of choosing the reduced risk option. Surprisingly, people who perceive their own health as below the average of people their age and gender are less likely to choose the reduced-risk option. This could capture respondents who have lower preferences for a healthy lifestyle. Gender, education, health insurance, and one’s own or a relative’s COVID-19 diagnosis have no statistically significant impact on WTP.

To illustrate the impact and relative magnitude of these determinants, marginal effects on the mean WTP are reported in the last column of Table 7. The marginal impact for a dummy variable was calculated by running the model twice, once with the indicator variable set to zero for all observations and a second time with the indicator variable set to 1, and then recording the change in the mean WTP. The WTP of people who have not been diagnosed with a chronic disease is 15 %, or $387, lower than the WTP of people who have a chronic disease. Respondents who believe their health is below average would be willing to pay 11 % or USD $279 less. The marginal effect for the continuous variables – risk reduction, logged income, and baseline risk – was calculated by running the model twice, once using the baseline model and a second time increasing the variable by the same amount for all observations. An increase in the risk reduction of 1 in 1,000 for each observation raises the mean WTP by 20 %, or USD $518. Increasing the baseline risk for each observation by 1 in 1,000 reduces the mean by a negligible $1. An increase in income of $500 per month for each observation raises the WTP by 3.6 %, or $94. Lastly, increasing the income of every observation by 1 % increases the mean WTP by $5.32, which is 0.2 % of the baseline mean WTP of $2,609, implying an income elasticity of 0.2.

The baseline estimation results are robust to different screening choices, as shown in Table 8. When excluding respondents who thought that the change in the risk was, the mean VSC equals $806,000, as in column 9. This is almost exactly the same as the baseline estimate of $805,000. Removing co-benefiters, respondents who considered changes in other health issues not described in the survey, slightly reduces the VSC to $796,000. When people who responded yes to both valuation questions and who indicated that they strongly agree that they would pay almost anything to reduce their risk (the yea-sayers) are removed from the estimation sample, the mean VSC equals $806,000. Excluding all people who indicated that they strongly agree that they would pay almost anything to reduce their risk, which is more restrictive than removing the yea-sayers, barely shifts the mean VSC to $805,000. Finally, removing protest responses, defined as those who strongly disagree that the survey gave sufficient information to decide or who did not answer as they would in real life, reduced the mean VSC to $804,000. Overall, the WTP and VSC estimates from these different screenings is effectively the same as the baseline estimate.

### Country-level estimates

The parametric estimation results of modeling each country separately are presented in Table 9. For all countries, the coefficients have signs and magnitudes that are consistent with the results from the baseline model. Scope sensitivity (i.e. the coefficient on risk reduction) is statistically significant for all countries, lowest in Germany and Türkiye, and highest in the United Kingdom and Chile. In all countries, the cost for the reduced risk option (i.e. scale sensitivity) has a negative effect on the probability to choose the reduced risk option and is statistically different from zero.

The highest country-specific mean WTP is $8,198 for China, which is a clear outlier. There are various factors that can explain such a high value. First, the median income of the sample of Chinese respondents is three times higher than the median income of the Chinese population, so the mean WTP estimated for China is biased upward, given the positive effect of income on WTP. Second, the health system in China likely incentivizes Chinese respondents to choose the reduced risk option, other things equal. Despite progress in recent years, China’s social security system provides limited protection, and medical bankruptcies pose a serious threat to many households. Insurance for sick leave and for healthcare costs is also uncommon; many Chinese respondents likely have limited or no sick pay and sick leave. In sum, having a chronic disease is more costly for individuals in China than for those in the other countries sampled where the health systems are more protective. When excluding China, the mean (median) WTP varies from $1,920 ($419) in the United Kingdom to $4,046 ($1,390) for Türkiye. The small median WTP for Canada is consistent with the high share of respondents who are indifferent to the valued item, shown by the large and statistically significant spike at zero.

Table 10 presents the estimation of country-level values using the pooled baseline model. This model includes post-stratification weights times country dummies, which not only corrects for the under- or over-representation of individuals in the sample compared to the actual population, but also captures “cultural” differences between the countries. It also increases the statistical power of the country-level estimate.

Country-level estimates of the mean WTP from the pooled baseline model are generated by recovering the individual-level WTP estimates for each observation. As described above, the individual mean WTP is computed by integrating the probability of an individual responding yes to the valuation question over the interval from 0 to the maximum bid, with an adjustment. The country-level mean WTP is the average of the individual mean WTP estimate for all observations from that country. The same approach is used for the median WTP and the VSC estimates.

The country-level results in Table 10 are generally consistent with the results in Table 9. A country-level estimate is not provided for China because it was not included in the baseline model. The largest impact of using the pooled model is on the estimate for Chile. The mean WTP and VSC increased by over 28 % to $3,163 and $994, respectively, and the median values decreased by over 20 % to $994 and $292. Most of the other changes in country-level estimates are less than 10 %, except for the VSC estimates for the U.S. The mean (median) WTP varies from USD $1,832 ($455) in the United Kingdom to $3,870 ($1,378) for Türkiye. The mean (median) VSC varies from $556,000 ($133,000) in the United Kingdom to $1,203,000 ($401,000) for Türkiye.

A bivariate regression indicates that GDP is a statistically significant determinant of cross-country differences in the mean VSC (*p*-value = 0.018, Adj. *R*^2^ = 0.51). However, preferences for reduced risk of serious kidney disease differ across countries and cannot be predicted by differences in GDP per capita alone. Other factors, such as differences in health systems, prevalence of kidney disease, and demographic factors, such as age distribution or cultural differences, might be more relevant, although these were not explicitly tested in this report. Overall, the significant variation in the mean VSC across countries illustrates why eliciting WTP values in various countries is important.

## 5. Conclusion

This study provides the first WTP estimates for pre-ESRD chronic kidney disease available for policy analysis, both directly in the surveyed countries, and through benefit transfer for others. Because preferences for health risk reductions can be expected to vary by country in ways that cannot fully be controlled for in the pooled values, country-specific estimates are more applicable for policy analysis. The country-specific values, with the exception of China, reflect the preferences of fairly representative sets of respondents in those countries. Consistency with the principles of benefit–cost analysis requires that benefits be valued as those who are affected would value them, and the country-specific estimates are the best reflection of preferences in each country.

The values developed here provide the basis for a more complete and rigorous characterization of the benefits of reduced risk of kidney disease. Importantly, these estimates are not limited to the “worst case” of kidney failure but are for symptomatic chronic kidney disease with the chance that it may result in kidney failure and premature mortality. As such, they are more representative of chronic kidney disease broadly, and more likely to be applicable for benefit–cost analysis of risk-reducing policies in many contexts, including chemicals regulation and food safety.

Looking forward, this study, in conjunction with the other surveys conducted under the SWACHE project, is likely to be informative for designing and implementing future cross-country surveys. For example, the survey shows that substantive survey revisions, in addition to translation verification, are likely to be necessary for a successful survey implementation in some countries. It may be the case that the results for China in this survey are due to language issues contributing to completion time anomalies, and more work may be needed for testing and refining the instrument after translating to mitigate such issues. In addition, as WTP estimates are systematically developed for more health endpoints in multiple countries, benefit-transfer both across countries and across health endpoints can be more fully evaluated.

## Figures and Tables

**Table 1.** Starting bids (total 5-year additional expenditure in USD)

Treatment group 1 (risk reduction of 2/1,000)			Treatment group 2 risk reduction of 5/1,000)		

Starting bid	Follow-up bid if starting bid rejected	Follow-up bid if starting bid approved	Starting bid	Follow-up bid if starting bid rejected	Follow-up bid if starting bid approved

300	150	600	600	300	1,200
600	300	1,200	1,200	600	2,400
1,200	600	2,400	2,400	1,200	4,800
2,400	1,200	4,800	4,800	2,400	9,600

**Table 2.** Sample quota versus achieved sample for chronic kidney disease survey

		Canada	Chile	China	Denmark	Germany	Italy	Norway	Türkiye	UK	U.S.

Gender											
Sample quota (%)	Male	50	49	51	50	49	49	50	49	49	49
	Female	50	51	49	50	51	51	50	51	51	51
Achieved sample (%)	Male	41	44	49	53	47	45	50	51	46	39
	Female	59	56	51	47	53	55	50	49	54	61
Age group											
Sample quota (%)	18–29	22	26	19	22	19	17	22	24	22	23
	30–44	28	31	31	25	25	27	28	34	27	27
	45–60	28	26	32	29	32	32	28	27	29	27
	60+	23	17	19	24	23	24	22	16	22	22
Achieved sample (%)	18–29	17	27	23	17	16	18	18	24	16	18
	30–44	25	35	37	18	26	27	28	41	27	28
	45–60	31	24	30	32	32	34	29	30	30	29
	60+	27	15	10	34	26	21	26	4	26	26
Level of Education											
Sample quota (%)	Low + Medium	41	75	86	60	70	80	56	78	53	52
	High	59	25	14	40	30	20	44	22	47	48
Achieved sample (%)	Low + Medium	49	71	81	56	69	79	48	62	50	57
	High	51	29	19	44	31	21	52	38	50	43
Median Monthly Income (2019)											
Sample quota	USD PPP	3,727	1,188	841	3,184	2,921	2,735	3,935	1,320	2,524	4,107
Achieved sample	USD PPP	3,706	1,744	2,754	3,562	3,692	2,671	4,563	3,457	3,338	3,751

**Table 3.** Response to first dichotomous choice question by starting bid

	Question 1: Per cent of respondents who chose to pay for the lower risk	
	No	Yes

Risk reduction = 2 / 1 000		
Starting bid = USD 300	6.39	6.20
Starting bid = USD 600	7.30	5.03
Starting bid = USD 1,200	8.22	4.46
Starting bid = USD 2,400	8.47	3.83
Risk reduction = 5 / 1 000		
Starting bid = USD 600	5.93	6.52
Starting bid = USD 1,200	6.66	5.70
Starting bid = USD 2,400	7.89	4.90
Starting bid = USD 4,800	8.66	3.84

**Table 4.** Summary statistics for continuous variables

Variable	Obs.	Mean	Median	Std. Dev.	Min	Max

Total time to complete the entire survey (minutes)	8,905	19.7	15.2	18.2	5.9	361.2
Time for 1st dichotomous choice question (seconds)	8,905	30.5	20	71.9	3	2,414
Time for 2nd dichotomous choice question if first response was No (Current risk)	3,609	14.5	8	135.2	2	5,993
Time for 2nd dichotomous choice question if first response was Yes (Lower risk)	5,309	13.3	8	153.6	2	11,032
Total time to complete both valuation questions (seconds)	8,905	44.3	30	163.4	12	11,041
Cost over 5 years for 1st dichotomous choice question (in USD)	8,905	1,680	1,208	1,390	267	4,900
Cost over 5 years for 2nd dichotomous choice question (½ or 2 times 1st cost)	8,905	1,740	1,153	2,014	133	9,800
Baseline Risk (/ 1 000)	8,905	29.4	25	17.3	15	60
Monthly household income (in USD)	8,030	4,068	3,290	3,122	263	17,552
Monthly household income (w/ predicted, in USD)	8,905	4,004	3,223	3,010	263	17,552

**Table 5.** Summary statistics for indicator variables

Variable	Obs, Variable = 1	Per cent of total obs. (%)	Variable	Obs, Variable = 1	Per cent of total obs. (%)

Canada	1,047	11.8	Respondents who said yes-yes to pay for risk reductions	2 233	25.1
Chile	981	11.0	Respondents who said no-no to pay for risk reductions	3 552	39.9
Denmark	939	10.5	Respondents who said yes-no to pay for risk reductions	1 372	15.4
Germany	1,031	11.6	Respondents who said no-yes to pay for risk reductions	1 748	19.6
Italy	1,022	11.5	Respondents with a high level of education	3 565	40.0
Norway	1,030	11.6	Health expenditures are out of respondent’s own pocket	1 226	13.8
Türkiye	872	9.8	Health perceived as below average or did not answer	1 416	15.9
U.K.	1,024	11.5	Health perceived as above average	3 449	38.7
USA	959	10.8	Relative or friend had kidney failure	3 095	34.8
Age 18–26	1,166	13.1	Respondents who have never been diagnosed with a chronic disease	5 857	65.8
Age 27–34	1,267	14.2	Respondents who have been diagnosed with COVID-19	757	8.5
Age 35–39	861	9.7	A close friend or family member was diagnosed with COVID-19	3 534	39.7
Age 40–44	888	10.0	Respondents who thought the risk reduction was permanent	514	5.8
Age 45–59	2,675	30.0	Respondents who considered other health issues (co-benefits)	820	9.2
Age 60–65	841	9.4	Respondents who strongly agreed they would pay almost anything	982	11.0
Age 65+	1,207	13.6	Yea Sayers: Answered yes-yes and strongly agreed to pay almost anything	554	6.2
Female	4,810	54.0	Protesters: Did not answer as in real life, or did not have enough information	301	3.4

**Table 6.** Main parametric estimations of WTP to avoid serious kidney disease

	Baseline	Without weights	No spike	Log-logistic	Log-normal	Including China	Excluding Türkiye
	1	2	3	4	5	6	7

Risk reduction (/ 1 000)	0.122*** (0.008)	0.122*** (0.008)	0.156*** (0.009)	0.165*** (0.013)	0.083*** (0.008)	0.121*** (0.008)	0.126*** (0.009)
Log(Cost)	−0.459*** (0.006)	−0.459*** (0.006)	−0.602*** (0.009)	−0.575*** (0.007)	−0.308*** (0.003)	−0.461*** (0.006)	−0.460*** (0.006)
Spike	0.035*** (0.002)	0.035*** (0.002)		0.024*** (0.001)	0.029*** (0.002)	0.031*** (0.001)	0.036*** (0.002)
Observations	8,905	8,905	8,905	8,905	8,905	9,709	8,033
Country dummies	No	Yes	No	No	No	No	No
Post-stratification weight x country dummies	Yes	No	Yes	Yes	Yes	Yes	Yes
Log-likelihood	−13,803	−13,800	−11,879	−14,196	−14,408	−14,778	−12,483
LR statistics	354	359	486	315	287	718	290
AIC	27,629	27,622	23,782	28,416	28,840	29,583	24,988
Mean WTP (USD)^°^	2,609	2,609	2,313	3,394	3,387	3,149	2,453
Median WTP (USD)	764	763	970	744	600	1,258	694
Mean VSC (K USD)^°^	805	805	698	1,082	1,093	983	753
Median VSC (K USD)	224	223	285	215	176	373	202

Note: The baseline estimation corresponds to a maximum likelihood estimation of the joint probabilities, assuming a Weibull distribution with a spike configuration. The baseline sample excludes survey responses from China. All columns exclude survey and valuation speeders as well as respondents who failed the risk tutorial test. Standard errors are given in parentheses. Signif. codes: 0

“***” 0.001

“**” 0.01

“*” 0.05 “+” 0.1.

° integral truncated at maximum bid level with adjustments.

**Table 7.** The determinants of WTP to avoid serious kidney disease

	Baseline	With controls	With health controls	
	Odd ratios	Odd ratios	Odd ratios	Marginal effect (USD)

Risk reduction (/ 1 000)	0.122*** (0.008)	0.123*** (0.008)	0.122*** (0.008)	518^°°^
Log(Income)		0.142*** (0.019)	0.134*** (0.019)	94^°°°^
Missing Income (0/1)		−0.029 (0.041)	−0.019 (0.042)	−74
Female (0/1)		−0.032 (0.027)	−0.028 (0.027)	−112
High education (0/1)		0.027 (0.028)	0.021 (0.028)	85
Baseline risk (/ 1 000)		0.000 (0.001)	0.000 (0.001)	−1^°°^
Health expenditure out of my pocket (0/1)			−0.042 (0.037)	−164
Health perceived below average (0/1)			−0.073* (0.037)	−279
Health perceived above average (0/1)			0.035 (0.028)	139
Relative had kidney failure (0/1)			0.043 (0.027)	173
Not diagnosed with chronic diseases (0/1)			−0.096*** (0.028)	−387
Was diagnosed with COVID-19 (0/1)			0.070 (0.046)	285
Relative was diagnosed with COVID-19 (0/1)			0.019 (0.026)	75
Log(Cost)	−0.459*** (0.006)	−0.460*** (0.006)	−0.460*** (0.006)	
Spike	0.035*** (0.002)	0.034*** (0.002)	0.034*** (0.002)	
Observations	8,905	8,905	8,905	
Country dummies	Yes	Yes	Yes	
Log-likelihood	−13,803	−13,767	−13,755	
LR statistics	354	426	450	
AIC	27,629	27,568	27,557	
Mean WTP (USD)^°^	2,609	2,633	2,637	
Median WTP (USD)	764	788	793	
Mean VSC (K USD)^°^	805	813	815	
Median VSC (K USD)	224	231	233	

Note: The baseline estimation corresponds to a maximum likelihood estimation of the joint probabilities, assuming a Weibull distribution with a spike configuration. The baseline sample excludes survey responses from China. All columns exclude survey and valuation speeders as well as respondents who failed the risk tutorial test. Standard errors are given in parentheses. Signif. codes: 0

“***” 0.001

“**” 0.01

“*” 0.05 “+” 0.1.

° integral truncated at maximum bid level with adjustments.

°° Marginal effects as a result of a1/1,000 increase in risk reduction or baseline risk reduction for each observation.

°°° Marginal effects as a result of a USD 500 per month increase in in baseline income for each observation. K means thousand.

**Table 8.** Robustness checks of the screening strategy

	Baseline	Excluding respondents who thought change was permanent	Excluding co-benefiters	Excluding yea- sayers	Excluding people who would pay anything	Excluding protest responses
	8	9	10	11	12	13

Risk reduction (/1,000)	0.122*** (0.008)	0.116*** (0.009)	0.124*** (0.009)	0.129*** (0.008)	0.127*** (0.009)	0.121*** (0.009)
Log(Cost)	−0.459*** (0.006)	−0.454*** (0.006)	−0.459*** (0.006)	−0 47*** (0.006)	−0.458*** (0.006)	−0.46*** (0.006)
Spike	0.035*** (0.002)	0.036*** (0.002)	0.035*** (0.002)	0.035*** (0.002)	0.038*** (0.002)	0.034*** (0.002)
Observations	8,905	8,391	8,085	8,351	7,923	8,604
Post-stratification weight *x* country dummies	Yes	Yes	Yes	Yes	Yes	Yes
Log-likelihood	−13,803	−13,029	−12,501	−13,111	−12,324	−13,319
LR statistics	354	313	326	332	316	334
AIC	27,629	26,082	25,026	26,245	24,671	26,661
Mean WTP (USD)^°^	2,609	2,604	2,563	2,156	2,238	2,605
Median WTP (USD)	764	746	749	603	607	764
Mean VSC (K USD)^°^	805	809	792	656	681	805
Median VSC (K USD)	224	220	219	175	176	224

Note: The baseline estimation corresponds to a maximum likelihood estimation of the joint probabilities, assuming a Weibull distribution with a spike configuration. The baseline sample excludes survey responses from China. All columns exclude survey and valuation speeders as well as respondents who failed the risk tutorial test. Standard errors are given in parentheses. Signif. codes: 0

“***” 0.001

“**” 0.01

“*” 0.05 “+” 0.1.

° integral truncated at maximum bid level with adjustments.

**Table 9.** Country-specific parametric estimations of WTP to avoid serious kidney disease

	Canada	Chile	China	Denmark	Germany	Italy	Norway	Türkiye	United Kingdom	United States

Risk reduction (/1,000)	0.127*** (0.024)	0.148*** (0.025)	0.107** (0.037)	0.140*** (0.026)	0.095*** (0.024)	0.144*** (0.026)	0.119*** (0.024)	0.087** (0.029)	0.147*** (0.024)	0.103*** (0.025)
Log(Cost)	−0.421*** (0.015)	−0.708*** (0.025)	−0.527*** (0.030)	−0.411*** (0.016)	−0.462*** (0.017)	—0.497*** (0.019)	−0.432*** (0.016)	−0.447*** (0.020)	−0.422*** (0.015)	−0.432*** (0.016)
Spike	0.049*** (0.006)	0.005*** (0.001)	0.008*** (0.002)	0.051*** (0.006)	0.035*** (0.005)	0.022*** (0.003)	0.043*** (0.005)	0.027*** (0.004)	0.056*** (0.006)	0.048*** (0.006)
Observations	1,047	981	804	939	1,031	1,022	1,030	872	1,024	959
Log-likelihood	−1,630	−1,518	−969	−1,458	−1,591	−1,506	−1,668	−1,314	−1,579	−1,445
LR statistics	27	42	12	32	18	33	24	10	38	17
AIC	3,267	3,043	1,946	2,924	3,191	3,020	3,343	2,636	3,167	2,899
Mean WTP (USD)^°^	2,350	2,462	8,198	2,552	2,297	3,067	2,536	4,046	1,920	2,143
Median WTP (USD)	552	1,253	5,019	611	638	1,080	646	1,390	419	496
Mean VSC (K USD)^°^	715	771	2,666	764	728	932	782	1,304	565	677
Median VSC (K USD)	158	387	1,552	170	194	311	188	426	116	149

Note: All models correspond toamaximum likelihood estimation of the joint probabilities, assuming a Weibull distribution with aspike configuration. All columns exclude survey and valuation speeders as well as respondents who failed the risk tutorial test. Standard errors are given in parentheses. Signif. codes: 0

“***” 0.001

“**” 0.01

“*” 0.05 “+” 0.1.

° integral truncated at maximum bid level with adjustments. K means thousand.

**Table 10.** Estimation of country-level WTP to avoid serious kidney disease using the pooled baseline model

	Canada	Chile	Denmark	Germany	Italy	Norway	Türkiye	United Kingdom	United States

Mean WTP (USD)°	2,277	3,163	2,329	2,358	3,233	2,468	3,870	1,832	2,120
Median WTP (USD)	612	984	631	642	1,016	684	1,378	455	555
Mean VSC (K USD)°	697	994	710	725	1,000	759	1,203	556	655
Median VSC (K USD)	179	292	183	188	297	200	401	133	164

## Footnotes

1 The OECD project Surveys on SWACHE brings together expertise on chemical safety and economic analysis to fill key valuation gaps. This unique collaboration involving partners and experts from many countries aims to establish internationally validated and comparable WTP values for several health outcomes associated with exposure to chemicals. The present study is one of the outcomes included in SWACHE. More information can be found at https://www.oecd.org/en/topics/sub-issues/risk-management-risk-reduction-and-sustainable-chemistry/the-costs-and-benefits-of-regulating-chemicals.html

2 Baseline risks were based on U.S. data and not adjusted by country in order to get values comparable across countries. One-on-one interview in non-USA countries show that using baseline risk based on U.S. data does not impact the credibility of the survey.

3 The bids presented to the respondents were converted from U.S. dollars into local currency using the OECD PPP for actual individual consumption adjustment for 2019. These values were then rounded to a whole number divisible by 10 to produce a value that was easy to understand by the local respondent. For the WTP estimation, the rounded values were converted back to USD using the PPP adjustment. Because of this rounding, bid values for the econometric analysis do not fall into the same discrete bins for all countries. Instead, bid values are a continuous variable.

4 See Dockins et al. (2023) for additional information on the Ipsos panel and Annex A specifically for the SWACHE core principles.

5 The outlier in the speeder identification analysis is China. While the Chinese country-level median time to complete the survey is close to the total sample median, China had an exceptionally large number of observations below 48% of the median. Screening for speeders removes 28% of the Chinese sample.

6 Equivalized income is the household’s total income from all sources divided by its equivalent size, calculated using the OECD-modified scale of 1 for the household head, 0.5 for each additional adult member, and 0.3 for each child. This adjusts income for differences in a household’s size and composition, reflecting the fact that household needs do not grow proportionately with each new member due to economies of scale in consumption. See https://www.oecd.org/els/soc/OECD-Note-EquivalenceScales.pdf and https://ec.europa.eu/eurostat/statistics-explained/index.php?title=Glossary:Equivalised_income for more information on equivalized income. 2019 was chosen because it was the last year of available data before the COVID-19 crisis.

7 The PPP data was extracted on 22 Feb 2021 08:44 UTC (GMT) from OECD.Stat, but has subsequently been revised. The exact series can be provided upon request.

8 Additional information on sampling weights is in Annex A of Dockins et al. (2023).
